# From arthritis to erectile dysfunction: potential pathophysiological mechanisms and multidisciplinary integrated management

**DOI:** 10.3389/fcell.2026.1729897

**Published:** 2026-03-27

**Authors:** Binbin Wang, Hongliang Cao, Shuxin Li, Zhanhao Li, Song Han, Zhijun Tang, Gengchen Huang, Yutao Ma, Bo Yuan, Wei Wei

**Affiliations:** 1 Department of Urology II, The First Hospital of Jilin University, Changchun, China; 2 Department of Spine Surgery, Center of Orthopedics, The First Hospital of Jilin University, Changchun, China

**Keywords:** ankylosing spondylitis, arthritis, endothelial dysfunction, erectile dysfunction, gout, inflammation, osteoarthritis, rheumatoid arthritis

## Abstract

This review systematically examines the underlying mechanisms linking erectile dysfunction (ED) with arthritis and explores multidisciplinary management strategies. Epidemiological studies confirm that patients with various arthritis—including osteoarthritis (OA), rheumatoid arthritis (RA), psoriatic arthritis (PsA), ankylosing spondylitis (AS), and gouty arthritis—exhibit significantly higher ED prevalence and incidence risks compared to the general population. This association remains independent of confounding factors such as age and comorbidities. Core mechanisms linking the two include chronic inflammation disrupting the “NO–cGMP–PKG” erectile molecular axis, vascular endothelial dysfunction causing insufficient penile blood supply, endocrine-metabolic disorders (e.g., insulin resistance, reduced testosterone), neuropsychological factors (anxiety, depression, chronic pain), and the synergistic effects of therapeutic agents (methotrexate, nonsteroidal anti-inflammatory drugs, glucocorticoids). Clinical recommendations advocate an “active screening-tiered assessment” approach, utilizing the International Index of Erectile Function (IIEF) combined with disease activity tools (e.g., DAS28, BASDAI) for screening and evaluation. Management should follow a multidisciplinary team (MDT) model integrating “control of underlying disease + individualized ED treatment + psychological intervention + lifestyle optimization” to improve patients’ overall health outcomes ultimately.

## 1 Introduction

Erectile dysfunction (ED) refers to a pathological condition in which men persistently or recurrently fail to achieve and/or maintain sufficient penile rigidity for satisfactory sexual intercourse (McCabe et al., 2016). As a highly prevalent sexual health disorder among men, its prevalence exceeds 50% in males aged 40–70 years (Saffati et al., 2025), exhibiting a significant age-related increase with further heightened risk in men over 70 (Wang et al., 2023). The etiology of ED is highly complex, resulting from the interplay of bio-psycho-social multidimensional factors (Hatzichristou et al., 2016). Clinically, it is categorized into three types: Psychogenic ED, triggered by psychological stress, emotional disorders, or interpersonal conflicts; Organic ED, further subdivided into neurogenic, hormonal, arterial, cavernosal (veno-occlusive), and drug-induced subtypes (Goyal et al., 2024); and Mixed ED, the most common clinical presentation due to the combined influence of organic pathology and psychological factors (Salonia et al., 2021). The current treatment framework for ED is relatively well-established, with phosphodiesterase type 5 inhibitors (PDE5Is) serving as the first-line therapy. Secondary interventions include intracavernosal injections, vacuum erection devices, and penile prosthesis implantation (Stern et al., 2025) (Nemr et al., 2025) (Lowy and Ramanathan, 2022). However, due to the sensitive nature of the topic, patients often avoid seeking medical help out of shame. Some healthcare providers may also hesitate to initiate discussions due to awkwardness or insufficient attention to patients’ sexual health needs. This results in low rates of spontaneous debate about sexual topics between doctors and patients, ultimately leading to persistently high rates of clinical underdiagnosis of ED (Miller, 2000) (Vasan et al., 2025). Notably, ED not only directly impacts patients’ sexual quality of life (QoL) but is also regarded as an “early warning signal” for male cardiovascular health, exhibiting close pathological associations with metabolic diseases like diabetes and hypertension (Yafi et al., 2016). Thus, achieving early diagnosis and proactive intervention for ED is essential for improving patients’ overall health outcomes.

Arthritis constitutes a complex group of diseases characterized by joint inflammation, encompassing over 100 subtypes. Common clinical forms include osteoarthritis (OA), rheumatoid arthritis (RA), psoriatic arthritis (PsA), gouty arthritis (Gout), ankylosing spondylitis (AS), and axial spondyloarthritis (axSpA) (Fallon et al., 2023) (Britto et al., 2025). Despite this diversity, all forms share common pathological manifestations: mononuclear cell infiltration, synovial inflammation and swelling, localized abscess formation, joint stiffness, and progressive cartilage destruction (Tang, 2019). Severe cases may progress to joint deformity or functional loss, significantly impacting patients’ physical and mental health and QoL (Pei et al., 2025). Epidemiological data indicate that arthritis exhibits a high global prevalence, affecting individuals across all age groups (with higher incidence among middle-aged and elderly populations) (Fallon et al., 2023) (Lites et al., 2023). Its long-term disabling effects and substantial healthcare expenditures constitute a significant socioeconomic burden. Clinical management of arthritis centers on “alleviating inflammation, controlling symptoms, and delaying progression.” Conventional interventions include nonsteroidal anti-inflammatory drugs (NSAIDs), disease-modifying antirheumatic drugs (DMARDs), and glucocorticoids, often combined with physical therapies like acupuncture and heat therapy for synergistic effects (Kolasinski et al., 2020). However, arthritis is not confined to localized joint lesions; it frequently involves multiple extra-articular complications spanning cardiovascular disease, metabolic disorders, and mental health abnormalities (Katz and Bartels, 2024) (Hin et al., 2024). Research confirms that approximately one-third of arthritis patients over 45 years old exhibit anxiety or depression symptoms, with anxiety occurring significantly more frequently than depression (Guglielmo et al., 2018). This state of psychosomatic comorbidity is a significant factor contributing to poor treatment response, chronic recurrence, and difficulty achieving complete remission. Crucially, the systemic chronic inflammatory response, vascular endothelial damage, mental health abnormalities, and metabolic disorders associated with arthritis represent key pathogenic mechanisms underlying ED. Recent clinical studies further confirm a significantly elevated comorbidity rate between ED and arthritis, suggesting a potential shared pathophysiological basis.

In clinical practice, ED is often overlooked due to its “private” nature, leading to long-term neglect of arthritis patients’ sexual health needs. Therefore, clarifying the characteristics and potential mechanisms associated with ED and arthritis and establishing targeted standardized screening and management protocols are of significant practical importance for comprehensively improving patients’ overall health outcomes. This review will systematically organize epidemiological evidence linking the two conditions, deeply explore potential pathophysiological mechanisms, analyze the intrinsic connections between their interactions, and outline future research directions. It aims to provide new insights for ED prevention and treatment while offering references for the clinical management of arthritis and related conditions.

## 2 Epidemiological evidence of erectile dysfunction and arthritis

Existing observational studies, cohort studies, and meta-analyzes consistently indicate that patients with various types of arthritis have a higher prevalence and incidence risk of ED compared to the general population. The strength of this association varies depending on disease type, disease activity, and comorbidities (Table 1).

**TABLE 1:** Main characteristics of epidemiological studies on arthritis and ED.

Study (Authors, year)	Disease	Study type	Number of cases (mean age in years)	Number of controls (mean age in years)	Diagnostic/Screening tool used	Main findings
Liu, C. et al. (2024) China	OA	Cross-sectional study	228 (60.0 ± 13.4)	2,851 (42.4 ± 14.8)	(NHANES)	(1) Among the different types of arthritis, OA was significantly associated with ED. (2) 39.4% of patients with arthritis developed ED, which was much greater than the 14.0% among those without arthritis
Yan et al. (2024) China	OA	Cross-sectional study	247 (NA)	3,123 (NA)	(NHANES)	(1) After fully adjusting for all potential confounders, OA showed a significant positive correlation with ED. (2) A remarkable ED prevalence of 16.56% among OA-diagnosed individuals, significantly surpassing the rates of 4.38% among non-OA counterparts and 7.33% within the NHANES cohort
Lamey et al. (2025) Egypt	OA	Cohort Study	100 (37.3 ± 9.72)	NA	IIEF-5	Egyptian obese males undergoing LSG demonstrated significant improvements in erectile function, osteoarthritis, and serum testosterone levels
Karabulut et al. (2022) Turkey	RA	Cohort Study	48 (45.58 ± 2.14)	NA	IIEF-5, DAS28, HAQ-DI	(1) After 6 months of tofacitinib treatment, the IIEF-5 score showed significant improvement (p < 0.001) (2) Changes in IIEF-5 scores were significantly correlated with reductions in DAS28 scores and improvements in HAQ-DI scores (p < 0.001)
El Miedany et al. (2012) Egypt	RA	Cross-sectional study	91 (51.4 + 9.4)	NA	PROMs, SHIM	(1) Among 91 male RA patients, the prevalence of ED reached 53.8% (2) ED significantly correlated (p < 0.01) with pain score, cardiovascular disease, age, disease activity, fatigue score, and tender joint count
Yan et al. (2024) China	RA	Cross-sectional study	182 (NA)	3,123 (NA)	(NHANES)	(1) After fully adjusting for all potential confounders, RA showed a significant positive correlation with ED. (2) The prevalence of ED among patients with RA was notably higher at 11.11%, markedly surpassing the rates of 3.82% observed among non-RA participants and the broader general population prevalence of 5.51%
Wilton et al. (2021a) America	RA	Cohort Study	260 (57.6 [SD 14.4])	260 (57.7 [SD 14.4])	REDCap	ED incidence was not increased in men with RA and ED diagnosis was not associated with an increased risk of cardiovascular disease in RA.
Wilton et al. (2021b) America	PsA	Cohort Study	128 (42.3 [SD 13.1])	128 (42.4 [SD 13.1])	CASPAR Criteria	(1) The cumulative incidence of ED in PsA was higher than the cumulative incidence in comparators, with an increasing divide between the men with PsA and the comparators with time (2) The 45% increased risk of ED in men with PsA compared to men without PsA did not reach statistical significance
Lobo et al. (2024) Brazil	PsA	Cross-sectional study	12 (52.1 ± 9.7)	NA	CASPAR Criteria, MSQ, IIEF	(1) Among the male patients, sexual performance was good to excellent (50%) or fair (50%) according to the MSQ. (2) Eleven of 12 patients (91.7%) had some degree of ED in at least one domain of the IIEF, with a predominance of mild ED.
Dhakad et al. (2015) India	AS	Case-control study	100 (age range: 20–56)	100 (age range: 20–50)	IIEF, HADS, BASFI, BASDAI, VAS, PGA	ED was associated with higher patient age, longer AS duration, anxiety, depression and higher BASFI in AS patients
Rostom et al. (2013) Morocco	AS	Cross-sectional study	110 (38.9 ± 12.5)	NA	BASDAI, BASFI, BASMI, HADS, Hamilton Anxiety Scale, MAF	(1) Thirty (41%) male AS patients reported experiencing ED. (2) Fatigue and sleep disturbance were independently associated with erectile dysfunction
Santana et al. (2017) Brazil	AS	Cross-sectional study	40 (45.8 ± 11.41)	40 (46.0 ± 11.1)	IIEF-5, BASDAI, HAQ, ASDAS	(1) AS patients had a median score on IIEF of 22.0, while controls had 29, with p < 0.0001 (2) Only 17.5% of the AS patients had no erectile dysfunction, in contrast to 87.5% of controls (p < 0.0001) (3) Multiple regression showed that BASDAI was the only variable independently associated with IIEF.
Nisihara et al. (2021) Brazil	AS	Cross-sectional study	35 (52.8 ± 7.1)	104 (51.9 ± 8.0)	IIEF-5, BASDAI, ASDAS-CRP	(1) AS patients had lower IIEF scores than controls (P = 0.02) (2) Among AS patients, IIEF scores showed significant negative correlations with BASDAI (P = 0.001) and ASDAS-CRP (P = 0.02)
Zhang Y. et al., (2023) China	AS	Meta analysis	393(NA)	NA	IIEF/IIEF-5, BASDAI	(1) The ED prevalence estimate was 44% with statistical heterogeneity (2) Men with AS were at a significantly higher risk for ED when compared with the general population without AS. (3) Patients with AS had significantly lower values in the IIEF erectile function domain as compared with the healthy control subjects
Fan et al. (2015) China	AS	Meta analysis	535(NA)	430(NA)	IIEF	Each domain of the IIEF score was lower in men with AS than in controls
Sariyildiz et al. (2013) Turkey	AS	Cross-sectional study	70 (36.4 ± 7.4)	60 (35.2 ± 7.7)	IIEF, BASDAI, BASFI, BASMI, BASRI, ASQoL, HADS	(1) The patients with AS had significantly lower scores in each of the 5 domains of the IIEF compared to the healthy control group (p < 0.05) (2) The BASDAI, BASFI, BASMI, BASRI, ASQoL, HADS scores and CRP levels were negatively correlated with IIEF (p < 0.05)
Erdem et al. (2020) Turkey	AS	Case-control study	50 (37.7 ± 7.6)	50 (37.0 ± 6.8)	IIEF-5, BDI, BAI, BASDAI, BASFI, BASMI, ASQoL	(1) The mean IIEF-EF domain score of the AS group was significantly lower than that of the control group (p = 0.004) (2) The mean IIEF score was lower in patients with AS, and this had a negative correlation with BASDAI, BASFI, ASQoL, BDI and BAI scores
Hu et al. (2025) China	r-axSpA	Cross-sectional study	113 (30.9 ± 7.3)	73 (29.1 ± 4.3)	SEQ, BASDAI, ASDAS, VAS, HADS	(1) Patients with r-axSpA scored significantly lower on erectile function score, individual satisfaction score and couple satisfaction score, and total score based on SEQ (p < 0.05) (2) The two-sample MR analysis demonstrated no causal effect of r-axSpA on the risk of ED based on the inverse variance weighted method
Leila et al. (2021) Tunisia	SpA, RA	Case-control study	50 (44.3 ± 12.1)	50 (48.8 ± 13.9)	IPSS, IIEF-5, BASDAI, ASDAS, BASFI	(1) The prevalence of ED in RD patients was not significantly higher than in controls (2) The severity of LUTS for patients with RD (RA and SpA) was significantly associated with ED (p = 0.008)
Abdul Sultan et al. (2017) England	Gout	Cohort Study	9,653(NA)	38,218(NA)	(CPRD)	(1) The absolute rate of ED post-gout diagnosis was 193 per 10,000 person-years. This corresponded to a 31% increased relative risk and 0.6% excess absolute risk compared to those without gout (2) Compared to those unexposed, the risk of ED was also high in the year before gout diagnosis
Hsu et al. (2015) China	Gout	Cohort Study	35,265 (49.6 [SD = 16.20])	70,529 (49.1 [SD = 16.50])	(NHIRD)	(1) Men with gout were more likely to have an increased risk of ED than those without gout (2) Patients with gout were 1.52 times more likely to develop OED and 1.18 times more likely to develop PED than patients in the control group (3) The risk of developing ED was greater for patients with comorbidities of CKD, diabetes, hyperlipidemia, depression, and anxiety
Chen et al. (2015) China	Gout	Cohort Study	19,368 (42.7 ± 12.0)	77,472 (42.7 ± 12.0)	(NHIRD)	(1) The gout cohort exhibited a 1.21-fold adjusted HR of subsequent ED development compared with the non-gout cohort (2) Compared to patients without gout and comorbidities, the patients with both gout and any type of comorbidity exhibited a 2.04-fold risk of developing ED.
Kim et al. (2019) Korea	Gout	Cross-sectional study	80 (52 [44–59])	70 (50 [42–55])	IIEF-5, HOMA-IR	(1) Gout patients were more likely to have ED than controls (2) HOMAIR was an independent predictor of ED.
Yigit et al. (2024) Turkey	Gout	Cross-sectional study	134 (56 [48–62])	104 (47 [40.5–54.5])	IIEF-EF, HOMA-IR	(1) The mean IIEF-EF score of gout patients was significantly lower than that of healthy subjects (2) Multivariable logistic regression revealed that increased CIMT was the only factor independently associated with ED.

Abbreviations: ED, erectile dysfunction; OA, osteoarthritis; RA, rheumatoid arthritis; PsA, psoriatic arthritis; AS, ankylosing spondylitis; SpA, spondyloarthritis; r-axSpA, rheumatoid factor-negative axial Spondyloarthritis; CASPAR, criteria, Classification of Psoriatic Arthritis Criteria; IIEF, international index of erectile function; IIEF-5, International Index of Erectile Function-5; IIEF-EF, International Index of Erectile Function-Erectile Function domain; SHIM, sexual health inventory for men; MSQ, male sexual quotient; SEQ, sexual experience questionnaire; NHANES, national health and nutrition examination survey; REDCap, Research Electronic Data Capture; CPRD, clinical practice research datalink; NHIRD, national health insurance research database; DAS28, Disease Activity Score in 28 Joints; HAQ-DI, Health Assessment Questionnaire-Disability Index; PROMs, Patient-Reported Outcome Measures; HADS, hospital anxiety and depression scale; BASFI, bath ankylosing spondylitis functional index; BASDAI, bath ankylosing spondylitis disease activity index; VAS, visual analog scale for pain; PGA, patient global assessment scale; MAF, multidimensional assessment of fatigue; BASMI, bath ankylosing spondylitis metrology index; ASDAS, ankylosing spondylitis disease activity score; ASDAS-CRP, Ankylosing Spondylitis Disease Activity Score-C-reactive Protein; BASRI, bath ankylosing spondylitis radiology index; ASQoL, ankylosing spondylitis quality of life questionnaire; IPSS, international prostate symptom score; HOMA-IR, homeostasis model assessment of insulin resistance; LSG, laparoscopic sleeve gastrectomy; NA, not available.

### 2.1 OA

OA is the most common degenerative joint disease, arising from the gradual wear and tear of articular cartilage over time. It can affect one or multiple synovial joints, including small joints of the hands and large joints such as the knees and hips. Primary symptoms of OA include joint pain, transient morning stiffness, and crepitus during joint movement. Ultimately, it may lead to joint instability and physical dysfunction, thereby impairing QoL (Martel-Pelletier et al., 2016). Traditionally viewed as a “wear-and-tear degenerative disease” caused by chronic joint overload and biomechanical abnormalities, OA has been primarily associated with cartilage destruction and subsequent inflammatory responses. However, it is now well-established that OA represents a complex pathophysiological process driven by both inflammatory mediators and metabolic abnormalities. This involves multi-tissue, multi-molecular coordinated regulation, rather than being solely the consequence of “mechanical injury” (Abramoff and Caldera, 2020).

Population studies demonstrate a significant association between OA and ED. An analysis based on the US National Health and Nutrition Examination Survey (NHANES 2001–2004) revealed markedly elevated ED risk among OA patients. The study showed an ED prevalence of 48.9% in the OA group *versus* 14.0% in the non-arthritis group (p < 0.001), indicating substantially higher ED incidence among OA patients. After fully adjusting for all potential confounders, OA remained significantly positively correlated with ED, with an OR of 1.11 (95% CI: 1.03–1.20, P = 0.017) (Liu C. et al., 2024). Another analysis based on NHANES 2001–2004 similarly demonstrated a significant positive correlation between OA and ED. The study showed that after fully adjusting for all potential confounders, OA patients still had a 113% increased risk of ED compared to non-OA patients (OR = 2.13, p < 0.0001). Suggesting the association between OA and ED is independent of other risk factors (Yan et al., 2024). Furthermore, an Egyptian follow-up study of 80 patients undergoing laparoscopic sleeve gastrectomy (LSG) found that among obese men with OA, the prevalence of OA decreased from 50.0% preoperatively to 18.8% 1 year postoperatively. Concurrently, ED-related indicators improved, with significant reductions in both subjective ED scores and objective hormonal markers (Lamey et al., 2025). Although this study did not directly analyze the independent association between OA and ED, it suggests that the relationship between OA and ED may be influenced by body weight status.

### 2.2 Autoimmune/inflammatory arthritis

Autoimmune/inflammatory arthritis (IA) constitute a group of chronic immune-mediated inflammatory conditions collectively termed IA. They are characterized by recurrent episodes of joint inflammation and progressive destruction of joint structures resulting from abnormal immune system activation (Chang and Nigrovic, 2019) (Noversa de Sousa et al., 2024). Clinically common forms of IA include RA, AS, PsA, and other forms of spinal arthritis (SpA) (Wu, 2020).

#### 2.2.1 RA

RA is a chronic, systemic, autoimmune inflammatory disease primarily affecting joints and surrounding soft tissues (Smolen et al., 2018). It leads to joint dysfunction, bone and cartilage damage, and may cause permanent functional impairment or disability (Behl et al., 2022) (Smolen et al., 2016). Most RA patients experience an insidious onset with slow progression, initially presenting as joint pain and swelling affecting one or a few joints, which gradually evolves into symmetrical polyarthritis (Di Matteo et al., 2023). RA can cause complications affecting multiple systems, many of which severely impact patients’ QoL and significantly increase mortality risk (Zeng et al., 2025).

Multiple studies have documented the association between RA and ED: A prospective Turkish survey of tofacitinib (a JAK inhibitor) in RA patients showed that among 48 patients with concomitant ED, the International Index of Erectile Function (IIEF-5) score increased from a baseline of 9.35–9.90 (P < 0.001) after 6 months of treatment. And the change in IIEF-5 score was significantly correlated with reduced disease activity score on 28 joints (DAS28) and improved QoL as measured by the Health Assessment Questionnaire-Disability Index (HAQ-DI) (p < 0.001), suggesting that controlling RA inflammation may improve ED (Karabulut et al., 2022). A cross-sectional study in Egypt involving 231 RA patients found that among the 91 male participants, the prevalence of ED reached 53.8%. ED was significantly associated with disease activity, pain scores, cardiovascular disease, and age (p < 0.01) (El Miedany et al., 2012). This suggests that male RA patients with higher disease activity, more severe joint pain, and concomitant cardiovascular disease have a higher risk and more severe degree of ED. A meta-analysis of 11 RA studies showed that in eight studies examining sexual function, the prevalence of ED/sexual dysfunction (SD) among male RA patients (33%–62%) was significantly higher than in healthy controls (11%–40%). This was similarly associated with disease activity and pain, and ED was frequently accompanied by decreased libido and reduced sexual intercourse satisfaction (Perez-Garcia et al., 2020). A cross-sectional study analyzing 3,305 participants further confirmed a strong positive correlation between ED and RA. This association remained significant even after fully adjusting for all potential confounders (OR = 1.67, 95% CI: 1.08–2.57, P = 0.0216), suggesting the relationship is independent of other risk factors (Yan et al., 2024).

However, existing research findings vary. An American cohort study (260 RA patients and 260 healthy controls) showed that the incidence of ED in RA patients did not differ significantly from age-matched controls and was unaffected by age stratification. However, RA patients with ED exhibited an increased risk of peripheral artery disease (PAD), with a significant interaction between RA and ED on PAD risk. Concurrently, these patients demonstrated reduced risks of myocardial infarction, heart failure, and mortality (Wilton et al., 2021a).

Current studies on the association between RA and ED exhibit varying results due to differences in geographic location, study design, and sample characteristics. Future research should involve larger sample sizes, multicenter settings, and long-term follow-up in prospective studies to clarify the differences in the association between the two conditions across different populations and identify key influencing factors.

#### 2.2.2 PsA

PsA is a chronic immune-mediated inflammatory disease and a seronegative inflammatory arthritis closely associated with psoriasis (Perez-Chada et al., 2025). Approximately 25%–30% of psoriasis patients develop PsA (Ganatra and Chandran, 2025) (Mehta et al., 2025). PsA can systematically affect joints, skin, and the musculoskeletal system, manifesting as inflammation-mediated multi-tissue damage (Feldkamp and Raychaudhuri, 2025) (Scrivo et al., 2023).

Two existing studies indicate a trend toward increased ED risk in male PsA patients: A U.S. cohort study of 128 age-matched pairs of PsA patients and controls showed a higher prevalence of pre-diagnosis ED in males (7%) compared to controls (3%) at baseline. And after long-term follow-up, the 20-year cumulative incidence of ED in the PsA group (19%) was also higher than that in the control group (13%), with PsA males having a 45% increased risk of ED compared to non-PsA males (Wilton et al., 2021b). Although the above comparison was not statistically significant, the gap in cumulative ED incidence between the two groups gradually widened with longer follow-up, suggesting a higher ED risk in PsA males. A Brazilian cross-sectional study involving 23 PsA patients found that 11 out of 12 male patients (91.7%) exhibited some degree of SD in at least one domain of the IIEF, predominantly mild SD. Additionally, the severity of psoriatic skin lesions (PASI) showed a significant positive correlation with patients’ overall sexual satisfaction, further supporting the association between PsA and increased ED risk in men (Lobo et al., 2024). However, existing studies are limited by small sample sizes and geographically concentrated populations. Future research should conduct multicenter, large-sample, cross-regional cohort studies to enhance statistical power through larger sample sizes and determine whether this association is statistically significant.

#### 2.2.3 AS

AS is a rheumatic and autoimmune disease associated with chronic, progressive inflammatory responses (Ni et al., 2024) (Zhang W. et al., 2023), primarily affecting the axial skeleton, such as the spine and sacroiliac joints, while also presenting with peripheral joint involvement and extra-articular manifestations (Martini et al., 2024). AS involves concurrent inflammation, bone erosion, and pathological ossification, disrupting the balance of bone remodeling (Feng et al., 2023). Clinically, it commonly manifests as pain, stiffness, or limited mobility in the spine and sacroiliac joints. Severe cases may lead to joint deformity, destruction, and even lifelong disability (Zhang W. et al., 2023).

A prospective case-control study involving 100 male AS patients and 100 healthy male controls demonstrated a significantly higher prevalence of ED in the AS group compared to healthy controls (OR = 3.04, 95% CI 1.52–6.13, P = 0.0006) (Dhakad et al., 2015), indicating a significantly higher risk of ED in AS patients compared to the healthy population. Another cross-sectional study involving 110 male AS patients found that 41% of sexually active male AS patients experienced ED, which was independently positively correlated with fatigue and sleep disorders (Rostom et al., 2013). A Brazilian cross-sectional study of 40 AS patients demonstrated that only 17.5% of the AS group had no ED, compared to 87.5% in the control group, representing a significant difference (p < 0.0001). Multivariate regression analysis further revealed that only the Bath Ankylosing Spondylitis Disease Activity Index (BASDAI) score was an independent predictor of ED (Santana et al., 2017), indicating that AS patients have a significantly higher ED prevalence than healthy individuals, with more severe ED associated with higher disease activity. A separate Brazilian cross-sectional observational study corroborated these findings. This study included 35 AS patients and 104 healthy controls, revealing that only 28.5% of AS patients reported “no ED” significantly lower than the 53.8% in the control group. Conversely, the “severe ED” rate reached 17.1% among AS patients, substantially higher than the 4.8% in controls. It also confirmed that AS patients overall had significantly weaker erectile function than healthy individuals, and ED showed a significant negative correlation with BASDAI and the Ankylosing Spondylitis Disease Activity Score - C-reactive protein (ASDAS-CRP) (Nisihara et al., 2021).

A meta-analysis incorporating eight studies and 393 adult male AS patients revealed an ED prevalence of 44%, indicating nearly half of AS patients experience ED. The study also confirmed that AS patients have a significantly higher risk of developing ED compared to healthy individuals, with a pooled relative risk (RR) = 2.04 (95% CI: 1.28–3.25, P = 0.003) (Zhang Y. et al., 2023). Another Chinese meta-analysis incorporating 11 studies reached similar conclusions. This study included 535 male AS patients and 430 controls. The results showed that AS patients scored significantly lower than controls across all IIEF dimensions, with the most significant difference observed in intercourse satisfaction (IS), followed by orgasmic function (OF) and erectile function (EF). Additionally, the incidence of ED and comprehensive sexual dysfunction was significantly higher among male AS patients than in healthy individuals, with a median prevalence of sexual dysfunction reaching 33.6%. This association was prevalent in both Caucasian and Asian populations (Fan et al., 2015). Another bicenter cross-sectional study involving 70 male AS patients and 60 healthy controls from rehabilitation departments at two Turkish university hospitals revealed significantly lower scores across all five domains of the IIEF compared to healthy controls (P < 0.0001). Further analysis indicated that BASDAI, the Ankylosing Spondylitis Functional Index (BASFI), the Ankylosing Spondylitis Measurement Index (BASMI), the Ankylosing Spondylitis Radiographic Index (BASRI), the Ankylosing Spondylitis Quality of Life Scale (ASQoL), the Hospital Anxiety and Depression Scale (HADS), and the inflammatory marker C-reactive protein (CRP) levels were all negatively correlated with IIEF scores (P < 0.05) (Sariyildiz et al., 2013). Additionally, a prospective case-control study in Turkey enrolled 50 male patients with confirmed AS and 50 healthy male controls. Results showed that the incidence of ED among AS patients (38%) was higher than that in healthy controls (30%), though the difference was not statistically significant. However, AS patients exhibited significantly lower IIEF-EF scores, with those experiencing ED demonstrating even lower scores (Erdem et al., 2020).

Collectively, these prospective case-control studies, cross-sectional studies, and meta-analyzes demonstrate that male AS patients exhibit a significantly higher overall risk and severity of ED compared to healthy males. Only one Turkish prospective study failed to observe a statistically significant difference in ED prevalence. Yet, it still confirmed significantly lower IIEF-EF scores in AS patients, further substantiating the negative impact of AS on male erectile function.

#### 2.2.4 SpA/axSpA

SpA is a group of chronic inflammatory rheumatic diseases often presenting with inflammation of the sacroiliac joints, peripheral joints, and spine, sharing overlapping genetic, clinical, and radiographic features (Lopalco et al., 2025; Rosine and Miceli-Richard, 2020; Toussirot and Wendling, 2005). AxSpA represents a subset of SpA primarily affecting the sacroiliac joints and spine, frequently progressing to ankylosis, severe disability, and functional impairment (Lopalco et al., 2025). Although chronic back pain and spinal stiffness are typical initial symptoms, peripheral (e.g., enthesitis, arthritis, and dactylitis) and extra-musculoskeletal (e.g., uveitis, inflammatory bowel disease, and psoriasis) manifestations are also common (Navarro-Compán et al., 2025) (Bittar and Deodhar, 2025). Based on the presence or absence of radiographic evidence of sacroiliac joint erosions and spinal ankylosis, axSpA can be classified as radiographic axSpA (r-axSpA) or non-radiographic axSpA (nr-axSpA) (Li et al., 2022) (McGonagle et al., 2024).

A Chinese cross-sectional study involving 113 male r-axSpA patients and 73 healthy male controls demonstrated that r-axSpA patients scored significantly lower than healthy controls on total sexual experience, erectile function, personal satisfaction, and partner satisfaction, with all differences being statistically significant (all p < 0.001). Multivariate linear regression analysis revealed that disease activity, physical disability, functional limitations, health status, sleep quality, and psychological state (anxiety/depression) were significant determinants of erectile dysfunction in r-axSpA patients (all p < 0.05). However, a two-sample Mendelian randomization (MR) analysis conducted as an extension of this study revealed no significant causal association between r-axSpA and ED risk (all p > 0.05) (Hu et al., 2025). These findings suggest that r-axSpA does not directly cause ED at the genetic level but somewhat indirectly mediates ED through disease-related symptoms and comorbid factors. Another cross-sectional case-control study involving 50 rheumatic disease (RD) patients (37 with SpA, 13 with RA) and 50 healthy controls showed an ED prevalence of 80% in the RD group *versus* 70% in healthy controls, with no statistically significant difference between groups (p = 0.2). Additionally, the study found that after stratifying patients based on the severity of lower urinary tract symptoms (LUTS) assessed by the International Prostate Symptom Score (IPSS) (mild/moderate/severe), the proportion of ED in RD patients showed a significant upward trend with increasing LUTS severity, with statistically significant differences between different LUTS severity groups (p = 0.03) (Leila et al., 2021). Although this study did not conclusively demonstrate that SpA directly increases ED prevalence, it suggests that RD may indirectly influence ED through other pathways. Further validation of these associations requires larger-scale longitudinal studies.

### 2.3 Gout

Gout is an inflammatory arthropathy caused by hyperuricemia, characterized by the deposition of urate crystals (monosodium urate, MSU) in joints and surrounding tissues, leading to acute inflammatory responses, pain, and swelling (Dehlin et al., 2020). Gout is marked by episodic acute inflammation, alternating between acute flare-ups and asymptomatic hyperuricemic periods (Helget and Mikuls, 2024), representing the terminal stage of uric acid (UA) metabolism disorders (Xu et al., 2025). The risk of gout increases with age, making it more prevalent in the elderly population (Dehlin et al., 2020).

Gout frequently coexists with metabolic syndrome. Existing research reveals a significant association between gout and ED, with gout patients exhibiting higher ED prevalence, incidence risk, and severity compared to non-gout individuals (Liu Y. F. et al., 2024). Studies indicate that elevated serum UA levels increase the risk of erectile dysfunction by more than 2.5-fold (Shen et al., 2025), and each 1 mg/dL increase in UA doubles the risk of ED (Salem et al., 2014). A prospective cohort study from the UK Clinical Practice Research Datalink (CPRD) revealed that among 9,653 gout patients and 38,218 controls, the incidence rate of ED in gout patients was 193 per 10,000 person-years, with a hazard ratio (HR) = 1.31 (95% CI: 1.24–1.40, P < 0.001), with an excess absolute risk of 0.6%. The study also found that ED risk was elevated 1 year before gout diagnosis (RR = 1.63, 95% CI 1.27–2.08) (Abdul Sultan et al., 2017).

A Taiwanese study based on the National Health Insurance Research Database (NHIRD), involving 35,265 gout patients and 70,529 controls, showed that gout patients had a 1.21-fold higher overall risk of ED compared to non-gout individuals. Specifically, the risk of organic ED (OED) increased by 1.52-fold, and the risk of psychogenic ED (PED) increased by 1.18-fold. The study also found that the risk of ED further increased when gout patients had comorbidities such as chronic kidney disease, diabetes, hyperlipidemia, depression, or anxiety (Hsu et al., 2015). Another nationwide cohort study based on the NHIRD reached similar conclusions. This study included 19,368 gout patients and 77,472 controls. Results showed that the incidence rate of ED in the gout cohort was significantly higher than that in the control cohort (p < 0.001). Additionally, the study found that gout patients with any one chronic disease (such as diabetes, hypertension, ischemic heart disease, *etc.*) had a significantly increased risk of ED (adjusted HR = 2.04, 95% CI: 1.63–2.57, P < 0.001). Furthermore, the incidence of ED in both cohorts increased with advancing age (Chen et al., 2015). A case-control study involving 80 gout patients and 70 healthy controls demonstrated a significantly higher prevalence of ED (55.3%) among gout patients compared to healthy controls (41.4%, p = 0.047). The study also indicated that gout patients had approximately 1.3 times higher ED prevalence than controls (Kim et al., 2019). Furthermore, gout may indirectly increase ED risk by affecting bilateral carotid intima-media thickness (CIMT). A cross-sectional study in Turkey involving 134 male gout patients and 104 healthy controls demonstrated that the gout group exhibited significantly higher prevalence of ED and incidence of CIMT thickening compared to the control group (both p < 0.001). Moreover, among patients with gout and CIMT thickening, the ED prevalence reached 97.9%, significantly higher than the other three subgroups (p < 0.001) (Yigit et al., 2024).

The above research evidence consistently indicates a clear association between gout and ED. The incidence and risk of ED in gout patients are significantly higher than in non-gout individuals. Elevated serum uric acid levels, coexisting T2DM, chronic kidney disease, hyperlipidemia, depression/anxiety, and CIMT thickening further exacerbate this risk. Notably, the risk of ED begins to rise as early as 1 year before a definitive gout diagnosis, providing a potential basis for early warning of ED in gout patients.

To summarize, epidemiological evidence across osteoarthritis, autoimmune/IA (including RA, PsA, AS, SpA/axSpA), and gout consistently shows that diverse arthritides are independently associated with increased prevalence and incidence of ED. This holds even after adjusting for confounding factors, including age, comorbid metabolic diseases, and cardiovascular conditions. This cross-subtype association prompts a central mechanistic inquiry: do inflammatory and metabolic arthritides converge on common final pathways to induce ED, such as shared vascular endothelial injury or disruption of the NO–cGMP–PKG axis? Or do their distinct primary pathologies impart unique mechanistic signatures that ultimately lead to erectile dysfunction? Examples of such distinct pathologies include proinflammatory cytokine dysregulation in rheumatoid arthritis, HLA-B27-mediated autoinflammation in ankylosing spondylitis, and hyperuricemia-induced cellular toxicity in gout. The following section will systematically dissect these potential pathways to address this critical question.

## 3 Potential mechanisms linking erectile dysfunction to arthritis

Although multiple observational studies have reported an association between arthritis and ED, and the incidence of ED is relatively high in such patients, the specific mechanisms remain complex and not fully elucidated. This section aims to explore potential pathways through which arthritis is closely associated with ED, centered on a unified pathophysiological axis: Arthritis (systemic inflammation/metabolic dysregulation) → vascular endothelial dysfunction and neuroendocrine imbalance → direct/indirect impairment of penile cavernosal smooth muscle and endothelial cell function → clinical ED. Within this axis, upstream systemic drivers (e.g., pro-inflammatory cytokines, hyperuricemia, insulin resistance) synergize to induce endothelial injury, which further disrupts cavernosal tissue homeostasis—processes supported by consistent observational and mechanistic evidence. Psychological factors and therapeutic drug effects modulate this core pathway, collectively increasing ED risk. These elements are interconnected rather than isolated, and understanding their hierarchical relationships is crucial for identifying novel preventive and therapeutic targets.

### 3.1 Chronic inflammation and vascular endothelial dysfunction

**FIGURE 1:**
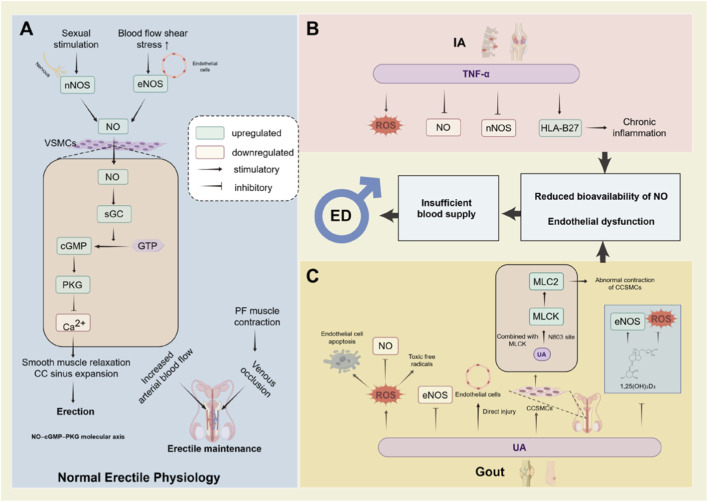
Normal erectile physiology and arthritis-induced ed pathological mechanism. This diagram shows the “NO–cGMP–PKG” axis (key for normal erection) and how arthritis may induce ED by disrupting this pathway *via* inflammation/endothelial dysfunction. Left Panel (A): Normal Erection. Sexual stimulation triggers NO production (mediated by nNOS/eNOS). NO activates sGC in VSMCs to produce cGMP, which then activates PKG. PKG reduces intracellular Ca^2+^, relaxes cavernous smooth muscle, and promotes penile arterial dilation/sinusoidal engorgement to complete erection. Right Panel: Arthritis-Induced ED. B (IA): Proinflammatory cytokines (e.g., TNF-α) increase ROS, suppress nNOS/eNOS, reduce NO, and damage endothelium. C (Gout): Hyperuricemia acts *via* four ways: ROS/NO depletion, eNOS inhibition, MLCK/MLC2-mediated abnormal contraction, and reduced active vitamin D. These processes collectively impair NO-cGMP signaling/endothelium, reducing penile blood supply and contributing to ED. Abbreviations: NO, nitric oxide; cGMP, cyclic guanosine monophosphate; PKG, protein kinase G; ED, erectile dysfunction; nNOS/eNOS, neuronal/endothelial nitric oxide synthase; sGC, soluble guanylate cyclase; VSMCs, vascular smooth muscle cells; CC, corpus cavernosum; CCSMCs, corpus cavernosum smooth muscle cells; Ca^2+^, calcium ion; TNF-α, tumor necrosis factor-α; ROS, reactive oxygen species; MLCK, myosin light chain kinase; MLC2, myosin light chain 2; GTP, guanosine triphosphate; PF, pelvic floor; UA, uric acid; HLA-B27, human leukocyte antigen B27; IA, inflammatory arthritis; Gout, gouty arthritis.

The core mechanism of normal erection relies on the nitric oxide (NO)-mediated “NO–cGMP–PKG” molecular axis, whose functional realization involves both neurogenic and endothelium-derived sources of NO (Jeremy et al., 2007) (Sheibani et al., 2022). Neurogenic NO is regulated by neuronal nitric oxide synthase (nNOS) and is produced following sexual arousal that activates non-adrenergic, non-cholinergic nerves. It rapidly induces penile vasodilation and sinusoidal engorgement. Endothelial NO, regulated by endothelial nitric oxide synthase (eNOS), is activated by penile blood flow shear stress, relaxing penile arteries and corpus cavernosum (CC) smooth muscle. This synergistic action, combined with the occlusive effect of pelvic floor (PF) muscle contraction on veins, collectively maintains erection (Meldrum et al., 2020). From a molecular pathway perspective, NO from any source diffuses into vascular smooth muscle cells (VSMCs) to activate soluble guanylate cyclase (sGC), promoting the conversion of guanosine triphosphate (GTP) to cyclic guanosine monophosphate (cGMP). Elevated cGMP further activates protein kinase G (PKG), which reduces intracellular calcium concentration by blocking sarcoplasmic reticulum calcium release, inhibiting calcium channel activity, and modulating potassium channels. This ultimately achieves smooth muscle relaxation, decreased penile arterial resistance, and expansion of cavernous sinuses, ensuring sufficient blood inflow to the corpus cavernosum and inducing erection (Jeremy et al., 2007). The etiology of ED is complex, with vascular ED being the most prevalent type. Endothelial dysfunction represents the primary pathophysiological mechanism underlying vascular ED, and any condition associated with arterial insufficiency or venous leakage can lead to erectile difficulties (Matsui et al., 2015) (Vlachopoulos et al., 2008). ED is generally recognized as a vascular disorder in which vasculitis plays a significant role. Previous studies indicate that compared to subjects without ED, ED patients exhibit elevated levels of tumor necrosis factor-α (TNF-α), interleukin-6 (IL-6), and interleukin-8 (IL-8), which correlate negatively with sexual health status (Liu D. et al., 2024) (Vl et al., 2006). These factors enter the circulatory system, inducing widespread endothelial damage that disrupts the normal physiological process of penile erection (Figure 1).

Arthritis is closely linked to disruptions of these erectile mechanisms through two core pathways: “chronic inflammation” and “vascular endothelial dysfunction”, with differing emphases across arthritis subtypes. IA conditions—such as RA, PsA, AS, and SpA—are characterized by systemic chronic inflammation (El Miedany et al., 2012) (Santana et al., 2017) (Roelsgaard et al., 2019) (Kurizky and Mota, 2012) (Perez-Garcia et al., 2021a). These diseases are associated with markedly elevated levels of proinflammatory cytokines such as TNF-α, IL-6, and IL-1β (Perez-Garcia et al., 2021a) (Kondo et al., 2021). On the one hand, TNF-α impairs endothelium-dependent vasodilation by increasing arterial reactive oxygen species (ROS) production, reducing NO levels, and simultaneously suppressing nNOS expression in the corpus cavernosum (Carneiro et al., 2010). On the other hand, in AS patients, TNF-α stimulation upregulates human leukocyte antigen B27 (HLA-B27) expression (Layh-Schmitt et al., 2013). HLA-B27 itself is prone to protein misfolding during processing (Colbert et al., 2014), and the aforementioned stimulatory effect of TNF-α further exacerbates this unfolded protein response. This results in patients being persistently in an activated unfolded protein response state, thereby inducing a proinflammatory state. Furthermore, HLA-B27 molecules can form dimers. When recognized by cell pattern recognition receptors, these dimers trigger autoinflammatory responses. The chronic inflammation induced by these processes may secondarily cause endothelial damage, which is ultimately associated with ED in AS patients (Chung et al., 2013).

Gout is primarily linked to vascular endothelium through hyperuricemia and metabolic disorders, which contribute to ED (Perez-Garcia et al., 2021b). At the molecular level, hyperuricemia induces endothelial damage by disrupting the balance between ROS and NO. UA production simultaneously generates ROS, which directly induces endothelial cell apoptosis, while interacting with NO to significantly reduce its bioavailability and generate additional endothelial-toxic free radicals (Gao et al., 2025). Additionally, elevated UA concentrations suppress eNOS activity and phosphorylation levels, further diminishing NO production at its site of synthesis (Nie et al., 2021), thereby doubly weakening NO-mediated vasodilation. Moreover, hyperuricemia impairs erectile function through the synergistic effects of “vascular structural damage” and “cavernous dysfunction.” On the one hand, UA directly damages vascular endothelial cells, triggering endothelial dysfunction that progressively evolves into atherosclerosis; arterial-origin ED represents a significant pathological subtype of ED, and atherosclerosis directly reduces penile arterial blood perfusion, blocking erection initiation at the level of blood supply (Chen et al., 2015). On the other hand, UA can penetrate the smooth muscle cell membrane of the corpus cavernosum, specifically binding to the N803 site of myosin light chain kinase (MLCK) within the cell. This binding inhibits the ubiquitin-mediated degradation of MLCK by the E3 ligase NEDD4L, leading to intracellular accumulation of MLCK, which in turn elevates the phosphorylation level of myosin light chain 2 (MLC2), ultimately triggering abnormal contraction of cavernous smooth muscle and disrupting the smooth muscle relaxation required for erection (Shen et al., 2025). Concurrently, hyperuricemia exacerbates endothelial dysfunction through multiple pathways, creating a cumulative injury effect. This includes inducing antiproliferative effects in endothelial cells, further increasing ROS production, reducing NO synthesis and utilization efficiency, and diminishing vascular responsiveness to acetylcholine, thereby comprehensively impairing vascular endothelial regulatory capacity (Yigit et al., 2024) (Du et al., 2016) (Kanellis and Kang, 2005). Furthermore, vitamin D deficiency is prevalent among patients with gout, and UA directly inhibits the synthesis of the active form of vitamin D (1,25(OH)_2_D_3_) (Yigit et al., 2024) (Luo et al., 2019). 1,25(OH)_2_D_3_ rapidly achieves non-genomic activation of eNOS through a vitamin D receptor-dependent phosphorylation cascade, thereby promoting NO production in endothelial cells (Andrukhova et al., 2014). Simultaneously, vitamin D inhibits superoxide anion production, blocking downstream pro-oxidative cascades to protect endothelial cells from oxidative stress damage and maintain endothelial function stability (Caretta et al., 2016). Studies indicate that vitamin D deficiency indirectly elevates ED risk by promoting endothelial dysfunction (Barassi et al., 2014), further linking metabolic dysregulation to endothelial injury in gout-related ED.

Overall, whether autoimmune or metabolic arthritis, the core mechanisms affecting ED center on “inflammation disrupting the NO pathway” and “endothelial dysfunction causing insufficient blood supply.” Targeted interventions, such as controlling UA levels and regulating inflammatory states, can effectively reduce the risk of ED.

### 3.2 Synergistic effects of endocrine and metabolic disorders

Endocrine-metabolic dysregulation, another critical upstream driver, synergizes with chronic inflammation to amplify endothelial damage and ED risk. ED is prevalent among patients with type 2 diabetes (T2D) (Blair et al., 2024). Multiple studies confirm that insulin resistance (IR) is a significant predictor of ED (Kim et al., 2019) (Russo et al., 2014) (Yao et al., 2013), with this association particularly pronounced in patients with gout. Insulin specifically activates the urate-anion exchanger (URAT1) on the brush border membrane of the proximal renal tubule. It may also activate the sodium-dependent anion transporter at this site, thereby enhancing renal urate reabsorption. Furthermore, under IR conditions, impaired oxidative phosphorylation leads to elevated intracellular levels of long-chain fatty acid-CoA esters, which stimulate systemic adenosine concentration, promoting UA production while simultaneously increasing renal reabsorption of sodium, UA, and water (Choi et al., 2007). This synergizes with insulin’s direct activation of transporters, collectively elevating serum UA levels. Studies indicate that for every one log pmol/L increase in fasting insulin, serum UA rises by 0.37 mg/dL (95% CI: 0.15 to 0.58, p = 0.001) (McCormick et al., 2021), forming a “metabolic dysregulation-inflammation-endothelial injury” cascade. Regarding testosterone levels, an analysis of 11 RA-related studies indicated that all three studies examining sexual function demonstrated lower total testosterone (TT) and free testosterone (FT) levels in RA patients compared to healthy controls (Perez-Garcia et al., 2020). Testosterone deficiency increases tissue oxidative stress, subsequently triggering endothelial dysfunction and ED (Pierre et al., 2022). Conversely, low testosterone levels further impact libido, penile tissue structure, and vascular function, exacerbating erectile dysfunction (Matsui et al., 2015). Men with hypogonadism exhibit elevated TNF-α, IL-6, and IL-1β concentrations due to impaired suppressive function, exacerbating endothelial dysfunction (Sansone et al., 2021) and creating a vicious cycle within the upstream driver network.

### 3.3 Potential impacts on physical and mental health and QoL

Arthritis influences the development and progression of ED by modulating the core “upstream drivers → end-organ damage” axis across multiple dimensions, including neurological, psychological, and physiological factors. These factors do not directly initiate the pathological cascade but amplify or mitigate its effects, becoming critical modulators of ED severity and prognosis.

Regarding neurogenic ED, the core mechanism involves damage to the neural pathways controlling erection, commonly seen in diabetic neuropathy, cortical injury, spinal cord trauma, or iatrogenic peripheral nerve injury from pelvic surgery (MacDonald and Burnett, 2021). A cross-sectional study in India also found that patients with RA exhibited significantly higher prevalence of autonomic neuropathy (AN), as measured both subjectively and objectively, compared to healthy volunteers. AN can manifest as ED (Aggarwal and Singla, 2017). Regarding psychogenic ED, psychological factors such as anxiety, depression, stress, and relationship issues can contribute to the condition by inhibiting the brain’s sexual response centers or increasing sympathetic nervous system activity (Matsui et al., 2015). Multiple studies confirm the strong association between anxiety and depression and ED. For instance, Sugimori et al. demonstrated significantly elevated ED risk in anxious patients aged 50–54 (OR = 2.48), while depression significantly increased ED risk in men aged 45–49 (OR = 3.42) and 50–54 (OR = 2.43). Patients with comorbid depression and anxiety in these age groups exhibited markedly higher ED prevalence than controls (Sugimori et al., 2005). A Turkish study revealed that Beck Depression Inventory (BDI) scores in male AS patients were significantly negatively correlated with both IIEF scores and IIEF classification (all P < 0.001), indicating that more severe depression correlates with more severe ED symptoms in male AS patients (Aykurt Karlıbel et al., 2019).

Furthermore, chronic pain and disability from arthritis undermine mental health, leading to decreased libido and worsening ED (Perez-Garcia et al., 2021b). Qualitative research indicates that feelings of frustration, shock, psychological stress, and diminished masculinity often accompany ED in men with IA. Anxiety about ED further exacerbates avoidance of sexual activity, creating a vicious cycle of “pain-ED-anxiety” (Restoux et al., 2020). Gout patients are similarly affected by psychological factors. Acute and chronic pain from gout, associated disability, depression, emotional distress, anger, and frustration can impact intimate relationships, thereby affecting sexual function (Singh, 2019). A case-control study based on the China Patient Records Database revealed that the cumulative probability of depression among gout patients was significantly higher than that of controls at 1, 2, 5, and 10 years of follow-up (p < 0.001 at all time points). After full adjustment, the adjusted HR for new-onset depression in gout patients was 1.19 (95% CI: 1.12–1.26, p < 0.05) (Kuo et al., 2016).

Regarding physiological ED, different arthritis impact erectile function through specific physiological mechanisms. Chronic pain in the spine or joints, fatigue in the evening or during disease flares, and limitations in position selection in patients with IA directly interfere with sexual activity, and fluctuations in disease activity can lead to recurrent ED symptoms (Restoux et al., 2020). AS restricts mobility in the axial skeleton (particularly the lower back), diminishing physical capacity for sexual activity. It is common pain (especially back pain) and loss of bodily function that directly impair sexual pleasure. Additionally, patients’ concerns about “back pain triggered by sexual activity” generate negative psychological states, further compromising sexual function (Fan et al., 2015). Studies indicate that, compared to AS men, those with morning stiffness lasting less than 2 h, those with stiffness exceeding 4 h exhibit lower erectile function scores (p < 0.05), suggesting that more prolonged and more severe morning stiffness correlates with poorer erectile function (Pirildar et al., 2004). OA patients face a bidirectional impact between sexual activity and disease. Poor sexual quality and low frequency negatively affect male erectile function on both physical and psychological levels. Conversely, those with poor erectile function may alter sexual positions to achieve satisfaction, and prolonged use of unsuitable positions increases joint wear, thereby elevating OA risk (Yan et al., 2024).

Notably, arthritis can also significantly diminish QoL due to psychophysiological factors like anxiety, depression, physical disability, and chronic pain, thereby increasing ED risk. A Taiwanese cross-sectional study involving 265 AS patients demonstrated a significant and independent association between higher AS disease activity and lower QoL (Lu et al., 2019). An Egyptian cross-sectional study showed a significant negative correlation between ED and QoL (p < 0.001) (Seyam et al., 2003). Another cross-sectional study reached similar conclusions, indicating a significant association between erectile function and QoL in male rheumatic disease patients (r = 0.257, p = 0.042) (Anyfanti et al., 2016). This suggests that ED severity may increase as QoL declines.

### 3.4 Potential effects of therapeutic drugs

In the treatment of arthritis, multiple core medications modulate the core “upstream drivers → end-organ damage” axis by directly interfering with physiological pathways or indirectly affecting bodily states, thereby increasing ED risk. These drugs act as modulators of the pathological cascade, either exacerbating end-organ damage or interfering with upstream driver control.

Methotrexate (MTX), as a first-line immunosuppressant for RA and other rheumatic diseases, has been clinically and mechanistically linked to induce ED. A Belgian cross-sectional study enrolled 77 patients with IA receiving MTX treatment and 32 controls. The results showed that patients with MTX use ≥5 years had significantly lower IIEF-5 total scores compared to those with <5 years of use and the control group (all P < 0.05). Furthermore, the IIEF-5 total score was negatively correlated with the duration of MTX use (r = −0.20, 95% CI: −0.38 to −0.04, P < 0.05) (Natalucci et al., 2024), indicating that longer treatment duration is associated with higher ED risk. Clinically, ED onset after MTX initiation ranges from 2 to 104 weeks (median 22.7 weeks), with recovery typically occurring within 2–12 weeks after discontinuation (Napolitano et al., 2024). Mechanistically, as a folate antagonist, MTX irreversibly binds and inhibits dihydrofolate reductase (DHFR) while also reducing mRNA transcription levels of the NOS gene (Napolitano et al., 2024) (Hanoodi and Mittal, 2025) (Papadopoulos and Papadimas, 2021). DHFR catalyzes both the conversion of dihydrofolate to tetrahydrofolate in folate metabolism and the reduction of dihydrobiopterin (BH_2_) to tetrahydrobiopterin (BH_4_); its inhibition reduces intracellular BH_4_ production. BH_4_ is an essential cofactor for NOS, and its deficiency triggers “NOS decoupling”—where NOS fails to synthesize NO using L-arginine as a substrate and instead generates ROS (Cronstein and Aune, 2020). This subsequently impairs vascular endothelial cell function or induces apoptosis, disrupting the physiological process of erection. Notably, the active folate form, 5-methyltetrahydrofolate (5-MTHF), stimulates NO production. It enhances NO bioavailability through multiple pathways: promoting BH4 regeneration, increasing BH4 bioavailability, upregulating DHFR activity, scavenging ROS, and directly binding to the purine site of eNOS (Stanhewicz and Kenney, 2017) (Sansone et al., 2018) offering a potential intervention strategy for improving MTX-related ED.

Nonsteroidal anti-inflammatory drugs (NSAIDs), as foundational symptomatic treatments for arthritis, are associated with increased ED risk (Gleason et al., 2011). NSAIDs inhibit prostaglandin synthesis by blocking arachidonic acid metabolism through cyclooxygenase (COX-2) inhibition, thereby interfering with NO synthesis and release (Li et al., 2018) (Li et al., 2023). A Finnish cohort study demonstrated that the incidence rate of ED among NSAIDs users was significantly higher than that among non-users. Even after adjusting for age, smoking, comorbidities such as diabetes/cardiovascular disease, and other medications, the incidence density ratio (IDR) for ED among NSAIDs users remained 1.8 (95% CI: 1.2–2.6), and the association between NSAIDs and ED was unaffected by the indication for use (Shiri et al., 2006). However, existing studies report conflicting evidence regarding the association between NSAIDs and ED (Li et al., 2018). One Chinese study employing two-sample Mendelian randomization (MR) analysis indicated that aspirin use may be associated with increased ED risk (Li et al., 2023). However, animal studies suggest that long-term aspirin administration does not affect erectile function in adult or aged rat models (Li et al., 2019). Furthermore, Darshan P Patel et al. found that both aspirin and non-aspirin NSAIDs were associated with increased ED risk. However, this study concluded that these associations were mainly attributable to confounding bias from indications, thus suggesting no association between NSAIDs use and ED risk (Patel et al., 2016). Consequently, further mechanistic research is needed to elucidate the complex relationship between NSAIDs and ED.

Glucocorticoids, commonly used in IA treatment, can affect erectile function through multiple pathways. They inhibit the production of the vasodilator NO and may interfere with choroidal blood flow regulation, thereby impacting penile rigidity (Hsu et al., 2015). Furthermore, glucocorticoids can influence erectile function by disrupting normal hormonal regulation. Long-term high-dose use may induce hypothalamic-pituitary-adrenal (HPA) axis dysfunction. Simultaneously, by interacting with glucocorticoid receptors on the testes, they can directly act on the gonads, collectively reducing testosterone levels (Østensen, 2017).

In summary, various medications used to treat arthritis are associated with an increased risk of ED through different mechanisms, and the potential effects of drugs should be emphasized in clinical treatment.

## 4 Clinical screening and assessment

Given the high prevalence and hidden nature of ED among patients with arthritis, it is recommended to establish an “active screening-stratified assessment” clinical pathway. Since ED patients often avoid proactive reporting due to feelings of shame, clinical practice requires combining subjective questionnaires with objective examinations to identify ED and its potential underlying causes comprehensively.

Among clinical screening and assessment questionnaires, the IIEF is a widely used tool for ED screening. The full-length IIEF-15 comprises 15 items covering five core dimensions of male sexual function, with EF as its central dimension. Based on EF scores (range 6–30), ED severity is classified into five grades: no ED (26–30 points), mild ED (22–25 points), mild-moderate ED (17–21 points), moderate ED (11–16 points), and severe ED (6–10 points) (Cappelleri et al., 1999). Clinically, the simplified IIEF-5 (scoring range 1–25) is more commonly used for ED screening and diagnosis. It similarly categorizes ED into five levels: no ED (22–25 points), mild ED (17–21 points), mild-moderate ED (12–16 points), moderate ED (8–11 points), and severe ED (1–7 points) (Rosen et al., 1999).​

Disease-specific tools should be selected based on the condition type to assess disease activity in arthritis. For example, DAS28 is used for RA patients (Karabulut et al., 2022); AS patients may utilize indicators such as BASDAI, BASFI, BASMI, ASDAS, and BASRI (Santana et al., 2017) (Nisihara et al., 2021) (Sariyildiz et al., 2013); Gout patients can be assessed by measuring serum UA levels (Shen et al., 2025) (Salem et al., 2014). These tools clarify the underlying disease’s activity and further elucidate the association between ED and disease activity.

It is important to note that patients with various arthritis conditions often experience comorbid psychosomatic symptoms such as depression, anxiety, and pain. These symptoms not only affect QoL but also increase the risk of developing ED. Therefore, clinical assessments should incorporate evaluations of these comorbid symptoms using tools such as the HADS, BDI, and Health Assessment Questionnaire Disability Index (HAQ-DI) (Karabulut et al., 2022) (Sugimori et al., 2005) (Aykurt Karlıbel et al., 2019) to evaluate depressive and anxiety states as well as joint-related functional impairment.

If questionnaire assessments indicate high disease activity, severe comorbid symptoms, or abnormal IIEF scores, heightened vigilance for ED risk is warranted. Referral to urology for objective evaluation is recommended to determine etiology or initiate early prevention. Among these, penile Doppler ultrasound (PDU) can identify the etiology and severity of vascular-related ED by measuring parameters such as peak systolic velocity (PSV), end-diastolic velocity (EDV), and vascular resistance index (RI) (Habous et al., 2024) (Gupta et al., 2015). With adequate arterial blood flow, PSV <25 cm/s indicates arterial insufficiency, while EDV >5 cm/s suggests venous occlusive dysfunction, enabling differentiation between these two etiologies of ED (Nashed et al., 2021). Furthermore, inflammatory joint diseases often coexist with endocrine and metabolic disorders. Assessing hormone levels and metabolic markers—including TT, FT, fasting blood glucose, and the insulin resistance index (HOMA-IR)—can aid in evaluating ED risk (Perez-Garcia et al., 2020) (Dilixiati et al., 2024) (Yao et al., 2013).

## 5 Treatment and multidisciplinary management strategies

**FIGURE 2:**
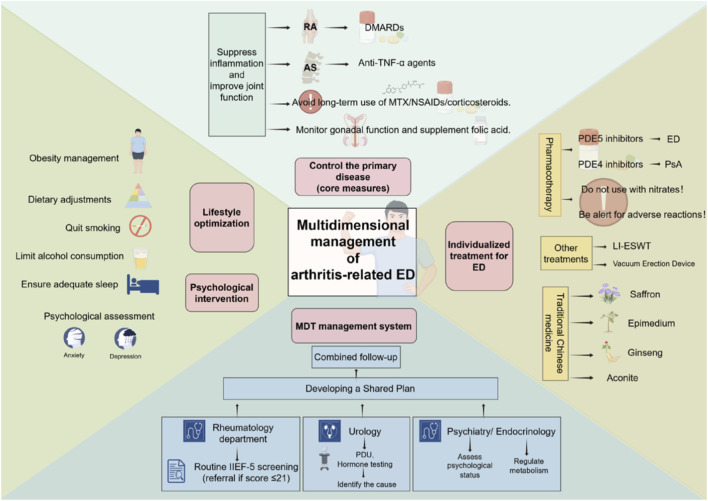
Multidimensional management strategies for arthritis-related ED. This diagram illustrates a comprehensive management strategy for ED associated with arthritis. Centered on the core dimensions of “controlling the underlying disease, individualized ED treatment, lifestyle optimization, and psychological intervention,” this strategy is implemented through a multidisciplinary collaborative management system to improve patients’ holistic health. Abbreviations: ED, erectile dysfunction; RA, rheumatoid arthritis; AS, ankylosing spondylitis; PsA, psoriatic arthritis; DMARDs, disease-modifying antirheumatic drugs; PDE5, phosphodiesterase type 5; PDE4, phosphodiesterase type 4; IIEF-5, International Index of Erectile Function-5; MDT: Multidisciplinary Team; LI-ESWT: Low-Intensity Extracorporeal Shock Wave Therapy; PDU: Penile Doppler Ultrasound.

The management of ED associated with arthritis employs a multidimensional strategy encompassing “control of the primary disease—individualized ED treatment—lifestyle optimization—psychological intervention.” Comprehensive patient health improvement is achieved through a rigorous multidisciplinary team (MDT) collaboration model that integrates expertise from rheumatology, urology, psychiatry, endocrinology, and clinical nutrition to address the complex interplay among systemic inflammation, local vascular injury, psychological distress, and metabolic dysregulation (Figure 2). This integrated approach ensures that interventions for arthritis and ED are synergistic rather than isolated, minimizing adverse effects and maximizing therapeutic efficacy.

Active control of the primary arthritis serves as the core therapeutic anchor, as suppressing systemic inflammation and improving joint function may indirectly mitigate ED progression. For RA patients, DMARDs or biologics with potential ED-beneficial effects should be prioritized. For example, tofacitinib—a selective JAK1/JAK3 inhibitor—reduces proinflammatory cytokine production by inhibiting STAT phosphorylation, alleviating RA inflammation, correlating with improved disease status, and mitigating ED severity (Karabulut et al., 2022). In AS patients, anti-TNF-α therapy for 3 months has been shown to improve disease activity, QoL, and erectile function significantly (Oh et al., 2009) (Dong et al., 2015). Emerging preclinical research has identified novel dual-targeting pyrazole derivatives (targeting COX-2 and the ACE1-N Domain) with potent anti-inflammatory properties, as well as the ability to increase serum NO levels and upregulate eNOS expression (Fadaly et al., 2024). This pharmacological profile provides a potential research direction for exploring future therapeutic strategies that may simultaneously address arthritis-related inflammation while preserving the NO-eNOS pathway, which is critical for erectile function. Additionally, a series of di-aryl-chalcone-derived pyrazole derivatives (16a-l) has been reported to exhibit enhanced COX-2 selectivity and anti-inflammatory activity in preclinical studies (Abd El-Hameed et al., 2025). Specifically, compounds 16d, 16f, 16k, and 16l exhibit higher COX-2 inhibitory potency and selectivity indices than celecoxib and can attenuate proinflammatory mediators, including PGE2, IL-6, TNF-α, and NF-κB (Abd El-Hameed et al., 2025). Given that traditional NSAIDs may interfere with NO synthesis and release by non-selectively inhibiting COX pathways (Li et al., 2018) (Li et al., 2023), these highly selective COX-2 inhibitors offer an alternative research direction for the management of arthritis symptoms. However, their efficacy in arthritis patients, as well as whether they can avoid the potential NO pathway disruption associated with traditional NSAIDs and thus influence ED risk, remains unaddressed and requires further investigation. Furthermore, long-term use of MTX, NSAIDs, and glucocorticoids should be avoided when possible; if clinically necessary, the rheumatology team should monitor gonadal function regularly and recommend folic acid supplementation to mitigate drug-related ED risks. For gout patients, urate-lowering agents to control serum uric acid levels are foundational, as hyperuricemia-induced endothelial damage and cavernous smooth muscle dysfunction are key mechanistic links to ED.

Individualized ED treatment should be tailored to the underlying etiology, with urologists leading this component of MDT care. As first-line pharmacotherapy for ED, PDE5 inhibitors exert their therapeutic effects by selectively inhibiting the PDE5 enzyme, which physiologically hydrolyzes cyclic guanosine monophosphate (cGMP) to 5′-guanosine monophosphate (5′-GMP); this inhibition prevents cGMP degradation, promotes intracellular cGMP accumulation, activates cGMP-dependent protein kinase (PKG), and subsequently reduces intracellular calcium (Ca^2+^) levels to induce smooth muscle relaxation *via* the NO/cGMP pathway (Nemr et al., 2025) (Padda and Tripp, 2025). Commonly used PDE5Is and their standard dosages include sildenafil (25–100 mg/day), tadalafil (5–20 mg/day), vardenafil (5–20 mg/day), and avanafil (50–200 mg/day) (Padda and Tripp, 2025). Despite their broad therapeutic applications, PDE5Is are strictly contraindicated in patients with specific severe comorbidities, including recent stroke or myocardial infarction, severe cardiac disorders (unstable angina, cardiac arrhythmia, and severe heart failure), uncontrolled hypertension, uncontrolled diabetes, and hypotension; concurrent administration with nitrates is also an absolute contraindication (Nemr et al., 2025). Case reports further note potential gout induction with sildenafil, warranting enhanced vigilance in patients with a history of gout (Alici et al., 2013). Immediate medical attention is mandatory if patients develop visual disturbances, hearing loss, or prolonged abnormal erections (Padda and Tripp, 2025). For complex refractory ED, combinations of low-intensity extracorporeal shock wave therapy (LI-ESWT) or vacuum erection device implantation may improve treatment efficacy (Mykoniatis et al., 2021). The urology team should further perform penile Doppler ultrasound (PDU) to distinguish arterial insufficiency (PSV <25 cm/s) from venous occlusive dysfunction (EDV >5 cm/s), thereby guiding targeted therapeutic interventions (Nashed et al., 2021). Additionally, for patients with PsA, the PDE4 inhibitor apremilast may provide dual benefits by ameliorating both joint inflammation and ED-related inflammatory pathways (Padda and Tripp, 2025).

Psychological intervention is a critical yet often overlooked component of MDT management, led by psychiatrists or clinical psychologists. Arthritis-related chronic pain, disability, and functional limitations frequently contribute to anxiety, depression, and sexual performance anxiety—factors that may amplify ED severity through a “pain-ED-anxiety” vicious cycle (Matsui et al., 2015) (Restoux et al., 2020). Routine screening with tools such as the HADS or BDI should be integrated into MDT assessments. CBT can help patients address negative thought patterns related to sexual function, while mindfulness-based stress reduction (MBSR) may alleviate anxiety associated with arthritis flares and ED (Bilal and Abbasi, 2020) (Sharpe et al., 2025). For patients with comorbid depression, selective serotonin reuptake inhibitors (SSRIs) should be prescribed cautiously, as these agents are known to cause sexual dysfunction, including ED and anorgasmia (Reisman, 2017); notably, some patients may experience persistent sexual symptoms after discontinuing SSRIs, termed SSRI-induced post-SSRI sexual dysfunction (PSSD), which presents as genital numbness, anorgasmia, or loss of libido (Healy and Mangin, 2024) (Bala et al., 2018). Thus, the psychiatric team should collaborate closely with urologists to balance mood management and sexual health.

Endocrinology and clinical nutrition support address metabolic and hormonal drivers of ED. Endocrinologists should monitor testosterone levels (TT and FT), insulin resistance (*via* homeostasis model assessment of insulin resistance, HOMA-IR), and glucose/lipid profiles, all of which are frequently dysregulated in arthritis patients and linked to ED (Perez-Garcia et al., 2020) (Dilixiati et al., 2024) (Yao et al., 2013). Testosterone replacement therapy may be considered for patients with documented hypogonadism, though its use should be balanced against potential cardiovascular risks and coordinated with rheumatology teams to avoid worsening inflammation. Clinical nutritionists play a key role in lifestyle optimization: obese patients should receive personalized weight management plans to reduce joint burden and improve metabolic health (Wang H. et al., 2025); gout patients require strict limitation of high-purine foods (organ meats, seafood) and alcohol; and all patients should be advised to increase omega-3 fatty acid (deep-sea fish, flaxseeds) and dietary fiber intake, while reducing high-sugar and high-fat foods to mitigate inflammation and endothelial dysfunction (Hong et al., 2024) (Bauer et al., 2020). All patients should also quit smoking, limit alcohol consumption, and ensure adequate sleep: Smoking induces oxidative stress and atherosclerosis in both active and passive smokers, increasing the risk of arthritis and ED (Siasos et al., 2014). Excessive alcohol consumption may lead to hypertension, while poor sleep quality elevates ED risk (Punjani et al., 2018).

To operationalize this MDT model, a structured clinical pathway should be established: Rheumatologists initiate ED screening using the IIEF-5 during routine arthritis visits, referring patients with scores ≤21 to urology for further evaluation. The urology team conducts PDU and hormone testing to identify ED etiology, while psychiatrists and endocrinologists complete concurrent assessments of psychological status and metabolic markers. A joint MDT consultation then develops a personalized plan that integrates arthritis treatment adjustments, ED pharmacotherapy, psychological support, and lifestyle modifications. Follow-up is coordinated every 3–6 months, with the MDT team reviewing IIEF-5 scores, disease activity metrics (DAS28, BASDAI, serum uric acid), and metabolic/psychological outcomes to refine interventions. Shared electronic medical records facilitate seamless communication across disciplines, ensuring that changes in arthritis management (e.g., medication adjustments) are promptly reflected in ED care and *vice versa*.

Complementary therapies may also be integrated into MDT management, provided they are carefully evaluated for evidence and safety. Chinese herbal medicines with anti-inflammatory and vascular-protective properties—such as saffron—can improve blood pressure, blood glucose levels, and inflammatory status and are widely used in cardiovascular diseases, diabetes, and RA, while demonstrating modest ED efficacy (Goyal et al., 2024). Epimedium, with pharmacological activities that enhance cardiovascular function and exhibit anti-inflammatory and antirheumatic effects, can be used to treat ED, RA, and AS (Qian et al., 2024) (Wang X. et al., 2025). Ginseng can prevent autoimmune processes in RA by inhibiting TNF-α and improve metabolic disorders by regulating the HPA axis. Regular intake may exert preventive and therapeutic effects on diabetes, RA, and ED (Lee and Rhee, 2017). Higenamine, a compound in Aconitum carmichaeli, exhibits vasorelaxant, anti-inflammatory, and antioxidant activities, demonstrating potential therapeutic value for arthritis and ED (Zhang et al., 2017).

In summary, the MDT model for arthritis-related ED transcends traditional siloed care by addressing the multifactorial nature of both conditions. By uniting rheumatology, urology, psychiatry, endocrinology, and clinical nutrition, this approach ensures that interventions target systemic inflammation, local vascular injury, psychological distress, and metabolic dysregulation simultaneously. Successful implementation requires structured screening, cross-disciplinary communication, and dynamic follow-up—ultimately improving both arthritis control and ED outcomes, and enhancing patients’ overall QoL.

## 6 Future research directions

To address existing research gaps and further clarify the intricate relationship between arthritis and ED, future studies should focus on specific, actionable avenues that integrate basic science, clinical translation, and clinical practice optimization, while adhering to cautious scientific inference.

First, mechanistic validation using disease-specific animal models tailored to the major arthritis subtypes may deepen understanding of local pathophysiological processes. Such models can be employed to systematically evaluate cavernosal hemodynamic parameters, including penile blood flow velocity and sinusoidal engorgement dynamics. Concurrently, *ex vivo* molecular analyzes of cavernosal tissue may explore the interactions between systemic inflammatory mediators (e.g., TNF-α, IL-6) and key local regulatory pathways, such as the NO–cGMP–PKG axis. This approach is anticipated to verify whether arthritis-associated systemic inflammation impairs the function of cavernosal smooth muscle and endothelial cells, thereby providing a molecular foundation for the development of potential targeted interventions.

Second, prospective clinical studies with long-term follow-up may validate the therapeutic relevance of anti-inflammatory strategies for ED improvement. Such studies could enroll patients with distinct arthritis subtypes and track changes in ED-related metrics (e.g., IIEF-5 scores) alongside specific anti-inflammatory therapies, including anti-TNF-α agents, JAK inhibitors, and urate-lowering drugs. Simultaneously, dynamic monitoring of serum biomarkers, such as inflammatory cytokines, endothelial function indicators, and metabolic markers, may enable correlational analysis between therapeutic responses, biomarker fluctuations, and ED improvement. This may clarify whether targeted suppression of arthritis-related inflammation can effectively mitigate ED, and identify subtype-specific therapeutic targets and response predictors.

Third, the development and validation of a clinical ED risk prediction tool tailored to arthritis patients may facilitate the early identification of high-risk individuals. Such a tool may incorporate key risk factors delineated in the present review, including arthritis disease activity, comorbidities associated with an elevated risk of ED, metabolic indicators linked to endocrine-metabolic dysregulation, and demographic characteristics (e.g., age, body mass index) consistently correlated with ED in arthritis cohorts. Multicenter, large-sample cohort data may be utilized to establish and internally validate the risk score, followed by external validation across diverse populations to ensure its generalizability. The tool should ideally be designed to be clinically feasible, enabling rheumatologists to efficiently evaluate ED risk during routine clinical encounters and facilitate timely screening or referral, thereby bridging the gap between arthritis management and sexual healthcare.

## 7 Conclusion

Observational studies, cohort analyzes, and meta-analyzes consistently confirm that patients with various arthritis subtypes (OA, RA, PsA, AS, SpA/axSpA, gout) are significantly more likely to develop ED than the general population, with this association remaining robust after adjusting for confounding factors such as age and metabolic or cardiovascular comorbidities. The unifying pathophysiological link centers on systemic inflammation and metabolic dysregulation, which may synergistically impair vascular endothelial function, disrupt the NO–cGMP–PKG axis, and induce cavernosal tissue damage. At the same time, neuropsychological distress, endocrine imbalances, and arthritis therapeutic adverse effects may further modulate ED risk. Clinically, proactive ED screening with tools such as the IIEF-5, along with integrated multidisciplinary management (combining arthritis control, individualized ED therapy, lifestyle optimization, and psychological support), is warranted. It is essential to acknowledge that while observational and mechanistic studies suggest plausible pathways, definitive causal relationships and the long-term efficacy of arthritis treatments on ED outcomes require validation through well-powered prospective and interventional trials. Recognizing ED as a common comorbidity of arthritis and addressing it via targeted assessment and collaborative care is essential to improving patients’ overall health outcomes and QoL.
